# Process evaluation of a pragmatic feasibility trial on smokeless tobacco cessation intervention delivered in dental hospitals

**DOI:** 10.1186/s12889-024-18821-2

**Published:** 2024-05-16

**Authors:** Shaista Rasool, Fiona Dobbie, Zohaib Khan, Richard Holliday, Fatima Khalid, Tuba Khan, Linda Bauld

**Affiliations:** 1 Usher Institute, University of Edinburgh, Edinburgh, Scotland; 2 Institute of Public Health and Social Sciences, Khyber Medical University, Peshawar, Pakistan; 3 School of Dental Sciences, Faculty of Medical Sciences, Newcastle University, Newcastle, England; 4 The University of Edinburgh, ACCORD, The Queen’s Medical Research Institute, 47 Little France Crescent, Edinburgh, EH16 4TJ UK

**Keywords:** Tobacco cessation, Smokeless tobacco, Dentists, Oral healthcare providers, Nawar

## Abstract

**Background:**

Article 14 of the WHO ‘Framework Convention on Tobacco Control’ recommends, that all oral healthcare providers provide support for tobacco cessation, to all patients. Despite evidence on the effectiveness of tobacco cessation interventions in dental settings, implementation remains low in most high-burden countries like Pakistan. A pragmatic pilot trial of a dentist-delivered behavioural support intervention for smokeless tobacco (ST) cessation, was conducted in dental hospitals in Pakistan. This paper presents the findings of the process evaluation of the trial.

**Methods:**

A mixed-method process evaluation of a multi-centre randomised control pilot trial of dentist-delivered behavioural support intervention ST cessation was conducted. The intervention included three sessions namely: pre-quit, quit and post-quit sessions. The process evaluation involved: semi-structured interviews with trial participants (*n* = 26, of which dental patients were *n* = 13 and participating dentists were *n* = 13 conducted from June-August 2022); and fidelity assessment of audio recordings of the intervention sessions (*n* = 29). The framework approach was used to thematically analyse the interview data.

**Results:**

Overall the trial procedures were well accepted, however, young patients expressed uneasiness over revealing their ST use status. The intervention was received positively by dentists and patients. Dentists identified some challenges in delivering behavioural support to their patients. Of these, some were related to the contents of the intervention whereas, others were related to the logistics of delivering the intervention in a clinical setting (such as workload and space). Acceptability of the intervention resources was overall low amongst young patients as they did not take the intervention resources home due to fear of their family members finding out about their ST use. The intervention was successful in achieving the intended impact (in those who engaged with the intervention), i.e., change in the patients’ ST use behaviour. Giving up ST with the aid of behavioural support also had an unintended negative effect i.e., the use of harmful substances (cannabis, cigarettes) to give up ST use. Patients’ satisfaction with their dental treatment seemed to influence the intervention outcome.

**Conclusion:**

While there are many variables to consider, but for the participants of this study, behavioural support for abstinence delivered through dentists during routine dental care, appears to be an acceptable and practical approach in helping patients give up ST use, in a country like Pakistan, where negligible support is offered to ST users.

**Supplementary Information:**

The online version contains supplementary material available at 10.1186/s12889-024-18821-2.

## Background

Smokeless tobacco (ST) products have been categorized as group 1 carcinogens by the International Agency for Research on Cancer (IARC) [1] and their use has been reported as the single most important risk factor in the all-cause mortality of oral cancer [2]. ST use is also associated with a higher risk of fatal heart diseases, adverse pregnancy outcomes and gastrointestinal disease [3]. Despite substantial evidence on the effectiveness of affordable tobacco cessation interventions, the progress in implementing Article 14 of the ‘Framework Convention on Tobacco Control’ (FCTC) (on tobacco cessation) has been slow, especially in lower-middle-income countries (LMICs), which share the greatest burden of ST use [4].

While many tobacco users wish to quit, the majority fail to do so. For instance, 51.% of smokers made a quit attempt in 2018 in the US, whereas only 7.5% successfully quit smoking [5]. Likewise, according to the latest Global Adult Tobacco Survey (GATS, 2014), 21.1% of the ST users in Pakistan made a quit attempt. In more than half of the cases, the quit attempt was un-assisted (50.8%). Dependence on tobacco makes it challenging to quit and cessation support is often minimal in most countries [4]. Treatments to aid tobacco cessation involve behavioural support, pharmacotherapies or a combination of these two approaches [6, 7]. Interventions to achieve behaviour change are complex and involve coordinated sets of interacting activities put together to influence and modify specified behaviour patterns [8, 10–13]. Behavioural support for tobacco cessation includes one or a combination of the following: advice; counselling; motivation and identification of strategies to cope with withdrawal symptoms [8]. As with other behavioural support interventions, there exists considerable variation in the content and delivery of behavioural interventions for tobacco cessation [9]. Typically, the interventions involve: offering advice to quit tobacco use; providing information on how to quit; or a combination of both. These interventions may be offered to tobacco users who are motivated to quit or to all users irrespective of their intention to quit. The intervention could be offered as a one-time brief advice or as more intensive, multiple sessions.

According to the latest Global Adult Survey (GATS 2014), the overall prevalence of tobacco use (smoked and smokeless) in Pakistan is 19.1% (12.4% smoked and 7.7% ST) [10]. The latest national figures indicate a rise in the use of ST (9%) (15% males and 3% females) [11]. ST use is deeply embedded in the social and cultural fabric of South Asia, where its use is widely associated with socializing, sharing and family tradition [12, 13]. In Pakistan, as in most South Asian (SA) countries, negligible cessation support is offered to ST users. One of the four key policy instruments identified in a recent study that explored ST control in Pakistan was ‘cessation services’ [14]. Whilst Pakistan, struggles to prioritise its funding and resources, it might not be possible to shift priorities [15]. However, the existing health system (such as dental settings), can be effectively engaged to extend its role to tobacco control with minimum investment. Every year more than 60% of tobacco users visit their oral healthcare providers (OHPs), which places them in a unique position to effectively contribute towards reducing the prevalence of tobacco use, whether that is by directing tobacco users to cessation services, or by engaging with patients in cessation counselling [16].

The limited literature on interventions for ST cessation and particularly behavioural support comes from research conducted in high-income countries, making the generalizability of such evidence to low-income settings challenging. There is a need for quality trials assessing the feasibility, efficacy and effectiveness of interventions for ST cessation in LMICs. An essential pre-requisite to definitive trials of the effectiveness and cost-effectiveness of behavioural interventions is a well-designed pilot trial, to address uncertainties regarding eligibility, recruitment and retention rates, and to explore the feasibility and acceptability of the intervention and the trial procedures. We, therefore, conducted a feasibility study involving a pragmatic randomised control pilot trial (*n* = 100) of a theory-based, dentist-delivered, behaviour change intervention for ST cessation in dental hospitals, in Pakistan. The findings of the trial are published elsewhere [17] and while the trial provided useful insights and points of consideration for future definitive trials and implementation, of equal importance was the need for a well-conducted process evaluation to strengthen the understanding of the implementation, receipt, and setting of the intervention. We have not been able to identify any ST cessation trial in dental settings which has reported process data. The dearth of such studies has constrained the development and implementation of complex ST cessation interventions, particularly in low-resource settings. The purpose of this study therefore was to conduct, a process evaluation of our trial of dentist-delivered ST cessation support. Besides considering what was delivered, the study aimed to understand how the intervention was delivered.

The UK Medical Research Council (MRC) provides a framework for the process evaluation of complex interventions and it involves the following key components: implementation (what is implemented and how); the identification of contextual factors (how does context affect implementation and outcomes? ) and mechanism of impact (how does the intervention produce change) [18–20]. The focus of process evaluation is generally determined by the stage at which it is conducted. At the feasibility and pilot phase, the focus is on understanding the feasibility of the intervention and also on understanding why interventions fail or succeed at this stage, to inform early design adaptations before progression into definitive trials. The process evaluation, which was embedded in the larger feasibility study, aimed to explore: the implementation of the intervention, identification of contextual factors and feasibility of trial procedures in line with UK MRC guidelines.

## Methods

### Overview of the pilot trial

A randomised control pilot trial, of a dentist-delivered behavioural support intervention for ST cessation was conducted at two dental hospitals in Pakistan. Details of the trial methods are published elsewhere [17, 21]. Briefly, 100 patients who were ST users were recruited at the two trial sites. All participants were given self-help written material on ST cessation. Whereas the participants in the intervention arm (*n* = 50), were offered behavioural support for ST. One-day training workshop was arranged for the dentists to train them on intervention delivery. The intervention was a structured behavioural support intervention for ST cessation, developed for users of South Asian (SA) origin ‘Behaviour Support for Smokeless Tobacco Users Of SA origin’ (BISCA) [16]. BISCA was delivered by dentists in three sessions namely; pre-quit, quit and post-quit. All sessions involved face-to-face counselling with the aid of a flipbook, which contained interactive messages for the participants to view on one side and prompts on the other side, for the dentists to guide the conversation with the patients. The trial was registered on the ‘International Standard Randomised Controlled Trial Number’ (ISRCTN) registry (ISRCTN18072109) on 13/01/2022.

### Process evaluation

A mixed-method process evaluation was conducted which involved: (a) semi-structured interviews with the trial participants (*n* = 26 of which 13 were patients and 13 were participating dentists); and (b) fidelity assessment through audio recordings of the intervention sessions (*n* = 29, 10 each from ‘pre-quit’ and ‘quit’ sessions, and nine from the post-quit session). Seventeen patients (out of the 50 randomised to the intervention arm) were invited for the interviews. Of these, 13 consented and were interviewed. Those who declined were out of town or could not take time off from their jobs.

The selection of patients for the interviews was based on self-reported quit rates in the trial and their engagement with the intervention. Of the thirteen patients interviewed, six patients self-reported abstinence at their third trial visit and seven reported continued use of ST. Furthermore, among the seven who reported continued use, two patients declined to attend the intervention sessions in the trial. The overall aim of the process evaluation was to explore the acceptability and feasibility of the behavioural support intervention for ST cessation. This was achieved through the following: (1) explore the views and experiences of the dental patients about the acceptability of a behavioural support intervention for ST cessation in routine dental practice; (2) explore the opinions and experiences of the participating dentists, regarding the implementation of a behavioural support intervention for ST cessation in routine practice; (3) explore the patients’ and dentists’ views and opinions about the trial processes; (4) quantify how much of the intervention components were delivered. The patients were given cash worth pkr1000 (GBP 2.83) to compensate for the transport cost to the interview. Details of the intervention are provided in (Additional file 1).

### Qualitative

The qualitative consultation with the trial participants (dentists and dental patients) was undertaken to explore the delivery and receipt of BISCA in a dental setting and; to explore the views of these two subgroups on the trial processes. Below is a description of the methods involved in this qualitative work.

### Interview topic guides

The topic guides enquired into the patients’ and dentists’ experiences regarding the intervention and trial procedures (Appendix 1 and 2).

### Participants

All dentists who participated in the trial were invited for the interviews. A non-probability sampling approach was adopted for purposively selecting patients from the intervention arm of the trial, to interview patients who: reported ‘abstinence’ to ST; continued using ST; belonged to different age groups and; had declined to attend the intervention sessions, while ensuring equal representation from both study sites.

### Study setting

The interviews with the dentists were conducted at the trial study sites (at their offices or the office of the head of department (HOD)). Interviews with the patients were conducted at either the trial sites or at Khyber Medical University (at the offices of dentists/faculty members), whichever was convenient for the patient.

### Procedure

Upon completion of the intervention sessions, patients in the intervention arm of the trial were contacted by the first author (SR) and invited for the interview. The dentists who delivered the intervention in the trial were invited face-to-face by SR during the hospital working hours.

All interviews were conducted in person by SR from June 2022 to August 2022. Privacy was observed for the interviews with only the interviewer and interviewees present during the interviews. Interviews were audio recorded and transcribed verbatim by SR. Reporting of the study methods have followed published standards for undertaking and reporting qualitative research (COREQ).

### Quantitative

Fidelity data was collected by audio-recording the interactions between the dentists and patients during the intervention sessions. The first ten counselling sessions delivered in the trial from each of the three sessions (pre-quit, quit and post-quit) were audio-recorded. The fidelity index consisted of 27 items and is provided in Appendix 3 [22]. Coding was done independantly by two coders and after coding each session, the two coders compared and discussed their scores, to develop a consensus on the scoring (agree upon a common score or retain the individual scoring). The definition of tailoring that was agreed upon for the assessment was ‘*where appropriate and needed, the dentist, tailors the intervention according to the patients’ needs e.g., the dentists recommending the use of “cardamom” for placing in the mouth instead of “chewing gum’’.* However, tailoring the intervention, to meet the patients’ socioeconomic needs, by skipping certain questions or topics, meant that the session was scored lower for that particular item, but scored high for tailoring. Index scores were summarized using mean and standard deviation and inter-coder reliability (percent agreement) of the item scores was computed.

The descriptive quantitative fidelity data was used to supplement the qualitative narrative, relating to intervention delivery (adherence to intervention protocol). To allow for a more insightful understanding of the delivery of the intervention components, methodological triangulation was performed by combining findings from the two qualitative data sources (patient and dentist interviews) and; quantitative assessment of fidelity to intervention delivery,

### Data analysis

Qualitative data was analysed thematically using the NVivo 12 software for data management. Rigorous line-by-line coding of all transcripts was done and data patterns were clustered into a thematic structure to identify and categorise major themes and sub-themes, which led to the development of the initial coding framework. This was jointly developed, piloted and amended by the members of the research team. This was followed by ordering and summarising of the data which led to data abstraction and interpretation involving organising the data under the main themes.

For the fidelity data, each recording of the intervention session was independently coded by two coders (SR and FK or TK). Each item was coded on a 3-point Likert scale (0 = not implemented, 1 = partially implemented, and 2 = fully implemented). Definitions of partial implementation were agreed upon for each item. The descriptive fidelity data enabled the assessment of fidelity to intervention delivery and contributed to the thematic analysis [23].

## Results

Of the 17 patients invited for interview, 13 consented and were interviewed. The average age of the patients was 41 years, with a range of 19–70 years. All patients were males. The ST product used by all participants was ‘naswar’ (the most popular ST product in Pakistan).

All dentists that were available (*n* = 13) were interviewed. Mean age of the dentists was 32.6 years with a range of 26 to 39 years, 38% (*n* = 5) were females and 62% (*n* = 8) were males. The duration of the interviews with the dentists ranged from 14.41 to 39 min Whereas the patient interviews ranged from 7.58 to 35.15 min. Results from the thematic analysis are discussed below under seven key themes, with illustrative quotes provided to supplement narrative descriptions.

### Theme 1: general acceptability of the BISCA intervention

There was generally a good commitment to the intervention amongst the dentists and patients. Dentists found the patients’ response positive and the experience of delivering cessation support rewarding, ‘different’ than what they had expected and different from their usual clinical work. For example, dentists anticipated uneasiness to approach the topic with their patients intervention but with hindsight, found patients receptive to the support, as the quote below illustrates.

*‘But some of our patients don’t listen to us at all, they say it’s my personal choice if I use illicit drugs or tobacco, they get offended if asked, so I expected that, but all patients were very responsive and participated fully.’ (Female, Dentist ID4)*.

Patients viewed face-to-face counselling by dentists in a private space as an acceptable and practical approach, however, they had varying experiences of the three sessions. While most patients shared positive accounts of the pre-quit sessions, their ‘quit’ session experiences were not all positive. The dissatisfaction expressed by a few patients was due to a lack of motivation/effort from the dentist, as narrated by a patient.

*‘She (the dentist) just asked if I use naswar and how much and it hardly took two minutes. It was very short. She didn’t tell me much in it. It wasn’t effective. I didn’t like it at all. The first session was so good. The dentist greeted me so well and counselled me in detail but in the second she just hurried through it’ (Patient ID2)*.

### Theme 2: adherence to the intervention

**Fig. 1:**
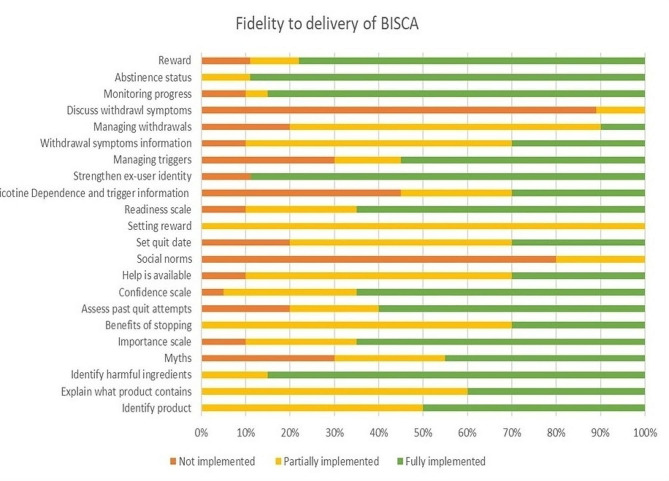
Percent implementation of the intervention components

Dentists failed to adhere to the intervention manual for certain components, as these were either partially implemented or not implemented. The percent implementation of the intervention components is depicted in Fig. 1.

The interview findings support this fidelity data. For instance, dentists found certain intervention components challenging to deliver. These included: ‘managing withdrawals’ and ‘managing triggers’, which involved helping the patients to identify strategies that would help them manage triggers and withdrawal symptoms. To this end, a dentist reported,

*‘Deep down inside me, I wasn’t fully convinced how chewing gum could help manage the urges of naswar. Some patients used naswar as an escape route to deal with stress related to financial and other problems, they said we use naswar to deal with stress or headaches.’ (Female, Dentist ID6*).

These challenges were augmented by the dentists’ lack of belief in the effectiveness of behavioural support and the absence of pharmacological aids. Reflecting on this a dentist stated,

*‘The socioeconomic status is so different here [compared to the U.K. where they had worked in the past], so if you ask someone to engage in activities that they find relaxing or to take their mind off their worries, most of them are so poor, the environment is so stressful here that people usually use naswar to help with the stress, so when they would say our friends use naswar, I used to find it very difficult to tell them not to use it’ (Female, Dentist ID11)*.

There was variability in how the contents were delivered, with the majority of the dentists placing more emphasis on the health harms, whereas, others focused more on the benefits of quitting. Information about withdrawal symptoms was reportedly provided in the pre-quit session instead of the quit session because dentists believed that informing patients about withdrawal symptoms in the first session was crucial in preparing them for the quit attempt, as is reflected in the following quote.

*‘I used to give them this information (about withdrawal symptoms) in the first visit. I think it should be in the first visit because we are preparing them to quit.’ (Male, Dentist ID1)*.

### Theme 3: acceptability of the intervention resources

Patients were given self-monitoring calendars in the quit session (for recording their daily abstinence status), which they had to return to the dentists in the post-quit session. Very few patients returned the calendars. Interestingly, younger patients did not take the calendar home due to fear of their family members finding out about naswar use.

*‘I didn’t take it home because my parents don’t know I use [naswar] so I didn’t take the calendar.’ (Patient ID12*).

Others who did not return the calendar either didn’t think it important to mark it or had lost it. Patients were also given a take-home flipbook in the pre-quit session, which contained images (and some text) providing a pictorial summary of the counselling sessions. Patients shared mixed views about it, as some patients found it helpful because it served as a reminder of the information provided in the counselling sessions. Others, however, did not go through the flipbook, as they did not believe in its role in helping them quit. As with the calendars, the younger patients did not take the flipbook home because they did not want to disclose their naswar use habit to their parents.

Overall, dentists found the use of the flipbook during the counselling sessions useful. The flipbook for the dentists had text for them on one side and images on the side facing the patient. It served as a useful prompt during the sessions helping the dentists to recall what to say next, in case they forgot. Reflecting on the use of the flipbook in the sessions, a dentist stated,

*‘It was very helpful because without it we didn’t remember most of the things. So we would be continuously taking help from it and it would remind us of any missed questions.’ (Female, Dentist ID3)*.

While dentists found the flipbook useful during the session, they had multiple suggestions to make it more effective. A common consensus was that the text on the flipbook for the dentists needs to be reduced, made more precise and eye-catching. They felt that it contained too much information which made it difficult for them to locate the text they were searching for.

### Theme 4: logistics of delivering BISCA

The intervention was delivered to dental patients at the largest teaching dental hospitals in the province, which share the greatest burden of dental patients in the region and deal with considerable resource constraints. The shortage of dentists at both of the study sites was viewed as a critical issue. The intervention increased the workload of the trainee medical officers (TMOs), as these were the ones most actively involved in the trial. Missed appointments and patients showing up on different days was an issue dentists faced as they would be busy with other patients when the trial patient would show up.

Senior dentists in the study facilitated the research team by helping arrange timely appointments for the trial participants, preferably with house officer (HO) or a TMO (rather than undergraduate students) within the time frame of the trial. Ensuring that the trial participant was assigned to an HO/TMO, was a problem identified by a senior dentist because instead of randomly assigning the trial patient to an undergraduate student (as would be done in routine) she had to ensure that the patient was assigned to a HO/TMO and one who was willing to treat the patient in the stipulated time. This meant the patient often had to wait longer to get the appointment. A separate room was identified for the counselling sessions. This room was the TMO’s tea room in one department, and the office of the HOD in the other departments. Whilst the dentists were overall satisfied with this arrangement, a few reportedly faced the problem of having to wait, in case the room was occupied. This at times led the dentists to deliver the intervention, in the ward or as one dentist mentioned to arrange for an alternate office while the patient waited.

*‘We had space issues because we used to counsel them in a separate room but that was someone’s office, so at times we had to wait.’ (Male, Dentist ID2)*.

Patients were overall satisfied with the space where they were offered counselling, as it offered privacy. The intervention sessions were scheduled alongside the patients’ dental appointments, i.e., the patients had to attend the intervention sessions on the day that they visited the hospital for their dental treatment. Overall, patients found such an arrangement acceptable. Some also felt that the reminders sent by the research team for the trial visits helped them keep up with their dental appointments, as otherwise they might have forgotten/not attended their dental appointments. Exceptions however existed, as one patient shared how he was in a hurry to leave the hospital due to his work and because he had given up naswar use, he did not feel like attending the remaining sessions.

*‘I thought everything was fine, my dental treatment was done, I have left naswar, and I had to rush back to work so I thought to miss the session’ (Patient ID3)*.

Likewise, an elderly patient who was visiting the hospital for a ‘complete denture’, reportedly felt annoyed by the interruptions in his treatment, presumably due to the intervention sessions. The department he was getting treatment from was under-resourced at the time of the trial in terms of dental units. Patients had to wait for a long time to get a vacant dental unit. He feared having to wait for the dental unit, in case he left the unit in the middle of the treatment (to attend the session).

*‘You know right that the chairs are few and everyone is waiting for a chair, so I asked the dentist to complete my work and then send me for counselling.’ (Patient ID9)*.

It is worthwhile to mention, that cases of complete dentures required lengthy appointments, with the dentist spending a substantial amount of appointment time in the laboratory working on the denture, while the patient remain seated in the dental chair. It was during this time (the time when the dentist was away in the laboratory) that the intervention was reportedly offered to the patient, thereby ensuring no interruptions in the treatment.

### Theme 5: dentists’ training

While overall the dentists found the training workshop helpful in increasing their knowledge, it wasn’t until they delivered their first sessions, that they learnt how to offer cessation support. Training involving actual patients who were ST users, rather than role plays was consistently identified as being a critical component that was missing. Commenting on the training workshop a dentist stated,

*‘The material that was given was very helpful and so was the workshop but the demonstration (role play), if it had been on a patient, it would have been better rather than a role play.’ (Male, Dentist ID2)*.

### Theme 6: perceived impact

All patients interviewed (except two) had either quit naswar or had reduced its use. The two patients who reported no change in their ST use behaviour, had attempted to give up naswar use after the intervention. One of them had declined to attend the ‘post quit’ session. He reportedly attempted to give up naswar use. However, due to the unsatisfactory experience with his dental treatment on the day of his quit session and the lack of support from the research team (to facilitate accordingly), he reportedly gave up on his quit attempt.

*‘It [counselling] was effective but because on that day my treatment plan was changed, I was so angry, that I stopped trying to quit naswar. I controlled myself a lot (didn’t use naswar) for a day or two after the second [quit] session but then I thought, they [research team] didn’t help me [with the dental treatment] so why should I try? I was so upset that I stopped trying.’ (Patient ID12)*.

The other patient needed naswar to manage a stressful event. Reflecting on his quit attempt he stated,

*‘I was miserable. I was in stress, I had headaches. I would have juice or chewing gum or candies etc. but I couldn’t manage. I couldn’t study at all. So I started using it again’. (Patient ID7)*

Offering patients behavioural support, changed the dentists’ perspective about the need for cessation support amongst naswar users and their willingness to quit. For example, one dentist stated,

*‘One thing I learnt was that patients want counselling, they need advice. Before the trial, I didn’t offer counselling to patients in routine, but now I try to counsel my patients. (Male, Dentist ID2)*

Despite the positive changes in the awareness about the need for support, there was only a slight change in the dentists’ clinical behaviour (taking ST use history, offering quit support). Multiple reasons were cited, which included; not being a habit (forgetting to take history); not being a protocol (worried that spending a lot of time on history taking might be frowned upon by other colleagues as it was not a routine protocol); lack of time (due to patient flow); lack of privacy and being a tobacco user (less common). Commenting on the inability to ask patients about ST use, a dentist stated,

*‘We are not expected to do this here, so I think that’s a big reason, If I ask about naswar, everyone will say, ‘Why is she asking and taking so long on the patient’, especially when other patients are waiting.’ (Female, Dentist ID11)*.

Some felt an inability to take history due to the fear of offending patients,

*‘I do feel hesitant in asking because it doesn’t look good you know, what if the person in front of me gets offended and what if he’s not willing to share.’ (Female, Dentist ID9)*.

A few of the dentists however had reportedly started offering brief advice at the chairside.

*‘I do ask about naswar and now I counsel everyone, I don’t take them to a separate room because making them leave the dental unit and taking them to another room isn’t easy, it needs a separate environment and privacy which is difficult in a clinical setting.’ (Female, Dentist ID4)*.

While the intervention was successful in achieving a positive change in ST use behaviour, it also had unintended positive effects such as patients passing on the cessation advice to other users, patients keeping up with their dental appointments and a positive impact on patient and dentist relation.

*‘Even now if we come across the trial patients, they connect with us, they feel that we are concerned about them, so they have more respect for us in their hearts now.’ (Male, Dentist ID12)*.

The intervention also caused a negative effect, which was the use of harmful substances to give up naswar use. These included the occasional use of cannabis and cigarettes to wean off of naswar.

*‘I didn’t use any strategy to quit naswar, I just threw it away and I would use date seeds to keep in my mouth or chew gum, but to be honest when I would feel sick, I would use cannabis.*’ *(Patient ID8)*.

The patients’ satisfaction with their dental treatment/hospital services during the trial period was likely to have an unintended positive or negative influence on the intervention outcome. For instance, as already mentioned above, patient declined to attend the third session and reportedly gave up trying to quit naswar due to the unpleasant experience related to his dental treatment.

### Theme 7: trial procedures: recruitment and retention

The process of recruitment via initial contact with the dentist (not involved in the trial), followed by a detailed discussion of the trial and its processes by the researchers was considered appropriate by the patients. They expressed satisfaction over how they were approached/identified and were reportedly comfortable disclosing their use of naswar to the dentist. There were, however, exceptions. For instance, a young patient did not like being invited to the study in the middle of ‘history taking’ and voiced concerns for other less educated patients. Sharing his experience he stated,

*‘They (dentists) were taking my oral history and asked me if I was interested in participating in a trial about ST. I mean I don’t have any complaints, I am studying to become a dentist myself too, but if they can approach me like this, imagine how they would approach a lay person.’(Patient ID5)*.

Another young patient also expressed dissatisfaction over how the history of ST use was taken. Sharing his experience, he narrated,

*‘There was a dentist there, she said something to you [researcher] which I didn’t like at all. She said, ‘Oh so now you will be interviewing naswar users huh?’ I felt so offended, I didn’t like the way she said it. It was as if she was mocking us and also everyone got to know that this person is a naswar user. It made me feel really bad.’ (Patient ID12)*.

It is interesting to note that the dissatisfaction over the ST use history was expressed by young patients only and all these young patients were educated, which reflects the growing social disapproval of naswar use within educated circles, as stated by a patient:

*‘My social circle includes educated people, so when I am in gatherings and parties, everyone looks down upon it, I feel ashamed of myself for using it.’ (Patient ID8)*.

The decision to participate in the trial for most patients was influenced by their desire to quit naswar. Other less common reasons included: a concern for the success of the dental treatment, curiosity and hope for freebies. Reflecting on his decision to participate, an elderly patient stated,

*‘I thought I’d get medicines but you didn’t give chocolates even (laughs).’ (Patient ID9)*.

The same patient mentioned the need for incentivising recruitment. Commenting on the lack of female representation in the trial sample, he stated,

*‘I know so many (female naswar users), you should have asked me, I would have helped you in recruitment. But you need to give them an incentive.’ (Patient ID9)*.

Assessment of the patients’ willingness to quit in the eligibility criteria was suggested by a dentist, who felt it was a waste of resources to offer structured behavioural support to patients who were not willing to give up ST uses. Narrating her experience in this regard she commented,

*‘But I do think that patients’ motivation to quit should be included in the recruitment criteria because this one patient I counselled for so long and he wasn’t willing to quit.’ (Female, Dentist ID6)*.

The motivation behind dentists’ participation included: interest in research, pressure from senior dentists and; interest in behaviour change.

## Discussion

This was the first study involving a mixed-method process evaluation of a trial on dentist-delivered behavioural support for ST cessation in an LMIC. While many studies have explored the factors that influence the implementation of tobacco cessation interventions in dental settings, there is limited evidence on the process evaluation of such studies in dental settings in low resource settings.

Overall, the dentists in the current study found it practical to deliver a behavioural support intervention in routine practice, however, certain issues were identified. Workload, time management and lack of privacy were some of the key issues and these factors have been frequently identified as barriers towards the implementation of tobacco cessation support within dental and other clinical settings, in previous literature [24]. For instance, a mixed-method implementation study assessing the feasibility of tobacco cessation, in a clinical (primary care) setting in Nepal, reported the issue of patients having to wait for long (up to an hour or were asked to visit the next day) to be counselled, due to workload/time availability [24].

Likewise, the infrastructure of the dental settings has also been reported in the literature as a barrier towards identifying users and offering tobacco cessation support. For instance, concern over the lack of privacy, due to the ward setting within the dental hospitals was reported in a more recent qualitative study exploring barriers towards ST cessation support in dental hospitals in a LMIC [25]. Likewise, the authors of another study, which assessed the feasibility of tobacco cessation in primary care settings, reported the potential contribution of the infrastructure of the clinical setting towards a low level of identification of tobacco users in the study [24]. The lack of privacy in the ward settings can undermine the practicality of the intervention in routine. While arranging a private space for taking a history of tobacco use, might not be practical in low-resource clinical settings, future trials should consider training the participating dentists on the skills required to take ST use history when no arrangements for privacy can be made.

Regarding the intervention delivery, most dentists in the current study did not deliver the intervention as planned. Literature on the dentists’ compliance with behavioural interventions for tobacco cessation is lacking, however, the findings of the current study are in keeping with the existing literature, regarding compliance with the protocol in non-pharmacological interventions [26–28]. For instance, in a multi-centre trial, Hovell et al. [28], reported that orthodontists did not deliver the tobacco cessation intervention as planned and that the anti-tobacco counselling was less seldom offered to the dental patients by the orthodontics in the intervention group. While the dentists’ non-compliance with the intervention delivery, could have been due to several reasons, the pragmatic approach to intervention delivery in our trial might have contributed towards the deviation from the protocol in intervention delivery. The dentists’ perceptions and beliefs and the patients’ responses and socioeconomic profile, might have influenced the delivery of the intervention. These findings are in line with the existing literature, as it is well established, that in addition to other factors (such as external, and organisational/contextual factors), how the individuals think, also influences the implementation of complex interventions [29, 30]. This brings us to one of the most debated topics in the translation of evidence-based health interventions, i.e., whether fidelity and adaptation can co-exist [30–32]. Behavioural interventions, among other things, are influenced by the socioeconomic needs of the patients, their responses and the beliefs and views of the provider. Therefore, given the adaptive nature of behavioural interventions, striking the right balance between flexibility in adherence (to allow for a pragmatic approach) and standardising the intervention, can be challenging, especially where tailoring is also a key quality element [32]. More research and a careful consideration are needed on the assessment of fidelity to the delivery of BISCA in clinical settings, especially for pragmatic trials (in which the intervention delivery is left at the discretion of the clinicians).

The dentists’ inability to implement certain items such as the ‘management of withdrawal symptoms and triggers’ were reportedly augmented by the perceptions of the dentists related the stress associated with the patients’ socioeconomic status (most naswar users belong to low socioeconomic background), the non-availability of resources to cope with stress in Pakistan, the absence of other cessation support/aids such as nicotine replacement therapies (NRTs) and the lack of cessation clinics in Pakistan. These findings support the existing literature on the barriers towards delivering tobacco cessation support in dental settings [33, 34]. Closely linked to this, are the findings from another pilot study that tested the same intervention (BISCA) in tobacco cessation clinics in Pakistan and England [22]. The cessation advisors in the study, reportedly felt that information on NRTs should be included in the intervention resources [22]. Future trials should therefore, consider including information about commercial nicotine pouches and NRTs in the intervention. The poor implementation of ‘self-reward’ in the current study, is also in keeping with the findings of the earlier BISCA pilot study [22]. Self-reward and self-incentives are techniques that are widely used in behaviour change, but little is known about the effectiveness of these techniques [35]. For instance, a systematic review exploring the effectiveness of these techniques did not find any study on self-reward, whereas the evidence in favour of self-incentives, was reportedly weak [35]. This raises the question of whether these techniques should be employed in behaviour change interventions and to whom and highlights the need for assessing the relevance of self-reward in ST users (and in all tobacco users) from SA.

The positive views expressed by the patients, about the dentist-delivered tobacco cessation support, support the existing literature [36–41]. Likewise, the lack of engagement of one patient with the intervention due to unsatisfactory experience related to the dental, also support the existing literature on the role of context, in that context cannot be divorced from the implementation of complex interventions [30].

The young adults’ reluctance towards tobacco cessation support (self-help material) as reported in the current study, has not been previously reported in literature. One reason could be that much of the literature on young adults’ tobacco cessation is largely from high-income countries [42]. The challenges faced by LMICs in the implementation of tobacco cessation interventions for young adults are perhaps yet to be explored, as they are specific to the sociocultural context of these countries. Future trials should consider the use of text messaging in lieu of take-home resources for younger participants, which they can use without the fear of social disapproval at home.

Regarding the change in the dentists’ clinical behaviour, the findings of the current study support the existing literature on the health professionals’ clinical behaviour with regard to tobacco cessation. For instance, in a pilot trial on tobacco cessation, Monson and Engeswick, observed no change in the dental hygienists’ behaviour of in provision of tobacco cessation support to their patients, after they had received training on the provision of tobacco cessation [43]. Similarly, according to the findings of a study investigating a pilot smoking cessation program in dental offices, none of the dentists had incorporated the tobacco cessation protocol, into their routine practice despite agreeing to do so, citing various reasons similar to what the dentists in the current study had identified [44]. Likewise, no significant impact on the patients’ receipt of tobacco cessation support was observed in another trial investigating a robust method for training clinicians in tobacco cessation [45]. The reasons identified by the dentists in the current study, for not being able to normalise the implementation of tobacco cessation support in routine included: lack of time; cessation support not being part of routine protocol; lack of privacy; fear of offending the patient and; use of tobacco among dentists. These findings are all in keeping with the existing literature [25, 44].

Overall, while much of the wider implementation of the intervention remains to be explored, and the need for a large-scale implementation study cannot be over-emphasized, the findings from the current study have provided some useful insights on the topic. To begin with, there is a need to invest in the capacity building of dentists. This would, among other things, require more emphasis on the treatment of tobacco dependence in the undergraduate curriculum for dentists and refresher training/workshops at the postgraduate level. Imparting the dentists with the skills and building their competence for recording tobacco use history, particularly the history of ST use, also needs attention, as this can positively contribute towards overcoming their reluctance to take ST use history. Other issues likely to be faced in routine implementation would be the lack of time and space. These would require ‘environmental restructuring’ through, for instance, allocating a quiet corner in the department (if not a separate room for the delivery of support). Policy-level support, from the administrative heads of the hospitals, will be required for the implementation of this measure.

Some limitations of the research are acknowledged. The main limitation of the study was that the interviews were conducted by the researchers who were involved in the trial. SR was known to all of the dentists and some of the patients (from previous contact at recruitment and follow-up within the trial). This might have influenced the dentists’ and patients’ responses. For instance, the dentists might not have openly shared their views on the training workshop, as it was facilitated by the research team. Likewise, the patients might not have felt comfortable openly criticising the conduct of the research. To address this issue, all participants (dentists and patients) were reassured before the interview that there are no right and wrong answers and that their responses were needed to improve the intervention and trial processes for future trials. Likewise, the positive change in the patients’ ST use behaviour, might reflect the effectiveness of the intervention or the influence the researcher had on the patients. While abstinence in the trial was verified by salivary cotinine analysis, the self-reported reduction in ST use was not verified. Therefore, the potential social desirability bias with the quit responses cannot be ignored. Another limitation was that the process evaluation did not take into account fidelity to intervention training and therefore relied only on fidelity to intervention delivery to understand non-adherence to the intervention protocol. An independent observation of the intervention training workshop would have strengthened the understanding of why dentists failed to adhere to the intervention protocol. While all participants who received the intervention should have been interviewed, given the time constraints, this was not possible and therefore a small, but diverse sample was selected, to understand the feasibility of the trial and intervention delivery in dental settings. No female patient was interviewed as only one female patient was recruited in the pilot trial and she was lost to follow-up. Furthermore, interviews with patients who were randomised to the control group in the trial were not conducted. Interviews with the control group could have offered a useful comparison between the views of the two groups.

## Conclusion

Dentist-delivered structured behavioural support intervention was found acceptable and perceived positively by dental patients and dentists. Workload and space were key challenges faced by dentists in delivering the intervention. The lack of pharmacological interventions and additional cessation support made it challenging for dentists to motivate patients to quit. Findings from this study could be used to inform future intervention development and optimisation as well as future implementation research in this area.

### Electronic supplementary material

Below is the link to the electronic supplementary material.

Supplementary Material 1

Supplementary Material 2

Supplementary Material 3

Supplementary Material 4

## Data Availability

The data generated and analysed during the study are available from the corresponding author upon reasonable request.
