# Supplementary material for: Process evaluation of a pragmatic feasibility trial on smokeless tobacco cessation intervention delivered in dental hospitals
Source: BMC Public Health. 2024 May 16;24:1327. doi: 10.1186/s12889-024-18821-2 (PMC11100072; doi:10.1186/s12889-024-18821-2)
Supplement: Supplementary file 1 — Supplementary Material 1 [file 12889_2024_18821_MOESM1_ESM.docx]

**Appendix 1: Topic guide (patients interview)**

**SECTION 1: EXPERIENCE OF BISCA**

1.1 OVERALL

1. Overall, how did you find the cessation sessions?

2. Were they what you expected? – What did you expect?

3. What did you like? –Why is that?

4. What did you dislike? - Why is that?

Talk through the sessions and activities in turn, using the flipbook

1.2 PRE-QUIT SESSION(S)

1. What did you discuss?

2. What did you learn? – was anything a surprise to you? If yes, what and why?

3. How helpful was this in preparing to quit? Why/why not?

4. What was most helpful?

5. At that time, how ready were you to set a quit date?

6. What helped you to feel ready/prevented you from feeling ready? (How useful was the prequit session in getting you ready?)

7. How confident were you that you could quit?

8. What helped you to feel confident/prevented you from feeling confident?

9. How useful was the pre-quit session in giving you confidence?

1.3 QUIT SESSION

1. What did you discuss?

2. What did you learn? – was anything a surprise to you? If yes, what and why?

3. How helpful was this in helping you to quit? Why/why not?

a. What was most helpful?

4. Did you make a plan for quitting using smokeless tobacco? What was your plan? If no, what

prevented you from making a plan?

5. Did you use the calendar? If yes, was it useful? How? If no, why is that?

6. At that time, how confident were you that you could quit from your quit date?

1.4 POST QUIT SESSION

1. What did you discuss?

2. How helpful was this in staying quit? Why/why not?

a. What was most helpful?

1.5. RESOURCES

1. Is there anything about any of these resources that you think we could improve?

2. Make them easier to understand? How?

3. Change the pictures/words? How?

4. What other pictures could we use?

**SECTION 2: PERCEIVED IMPACT OF BISCA**

Since completing your cessation sessions, have you managed to quit using smokeless tobacco?

If yes

1. Why did you quit?

2. How did you manage to do it? What strategies did you use?

3. What are the benefits of you quitting smokeless tobacco? to you? to your family? to anyone

else?

4. Are there any disadvantages? Please tell me.

If no, but have reduced use; what has changed?

1. When did you reduce your use? By how much?

2. How did you manage to do it? What strategies did you use?

3. What are the benefits of you reducing your use of smokeless tobacco? to you? to your family?

to anyone else?

4. Of these, which is most important to you? Why is that?

5. Are there any disadvantages? Please tell me.

6. Do you plan to try to quit? - When? Or why not?

If no change, why do you think that was?

1. What barriers did you face? How did you try to overcome them?

2. Will you try to quit in the future? When? What will you do differently?

3. If not, why not?

4. Is there anything else you want to tell us about the cessation programme?

**SECTION 3: STUDY PROCEDURES**

1. How did you learn about this study? What other ways could we tell people about the study?

Different places? Written or verbal information? Why is that a good idea?

2. What information about the study did you receive? How useful was it? How can we make it

more useful? What else do we need to say?

3. Is there anything else that you want to say?
