# Supplementary material for: Process evaluation of a pragmatic feasibility trial on smokeless tobacco cessation intervention delivered in dental hospitals
Source: BMC Public Health. 2024 May 16;24:1327. doi: 10.1186/s12889-024-18821-2 (PMC11100072; doi:10.1186/s12889-024-18821-2)
Supplement: Supplementary file 2 — Supplementary Material 2 [file 12889_2024_18821_MOESM2_ESM.docx]

**Appendix 2: Topic guide (dentist interview)**

**SECTION 1: EXPERIENCE OF BISCA**

1.1. OVERALL

1. Overall, how did you find the cessation sessions?

2. Were they what you expected? – what did you expect?

3. What did you like? –Why is that?

4. What did you dislike? - Why is that?

4.5PRE-QUIT SESSION(S)

1. How many pre-quit sessions did you deliver?

2. What did you discuss?

3. How helpful do you think this was in preparing your patient to quit? Why/why not?

4. What was most helpful?

5. At that time, how ready do you think your patients were to set a quit date?

6. How useful do you think the pre-quit session was in getting your patients ready?

7. How confident/prepared were you that you could deliver the intervention effectively?

8. What helped you to feel confident/prevented you from feeling confident?

9. How useful was the training workshop in giving you confidence?

4.6QUIT SESSION

1. What did you discuss?

2. How helpful was this in helping your patients to quit? Why/why not?

a. What was most helpful?

4.7POST QUIT SESSION(S)

1. How many post-quit sessions did you deliver?

2. What did you learn? – was anything a surprise to you? If yes, what and why?

3. How helpful do you think this was in helping your patients remain abstinent ? Why/why not?

a. What was most helpful?

1.5. RESOURCES

1. Can you please describe how you were using the flip book?

2. How did you feel about using the flip book?

3. How did you feel about the images?

4. How did you feel about the texts on the back: too much text, not enough text? How do you

feel about the size and dimensions of the book: is it easy to hold and use during a

consultation?

5. How do you feel about the tobacco cessation messages in the flip book?

What is the reason for this?

1.6. PATIENTS’ RECEPTIVNESS

1. How have patients responded to the flip book and the tobacco cessation messages?

2. Are there any differences between patients from different backgrounds?

3. (Rural/urban, highly educated, not well educated, with different occupations, of different

age, with different illnesses other than dental, specific illnesses: who are hearing/visually

impaired)

4. How did you use the materials with patients who were illiterate? Can you describe what you

said or did differently – if at all – from your other patients?

5. (Did you use the flip book differently with patients who couldn’t read?

6. How have patients responded specifically to the tobacco information and advice you gave?

7. Are there any differences between patients from different backgrounds? What have

patients said about potential difficulties stopping tobacco?

8. What were they worried about?

9. (Tobacco use in their families, among their friends, at work, in social situations afraid of

quitting or side effects, not knowing how to quit.)

1.8. INTEGRATING IN ROUTINE, CONTEXT

1. How do you feel about delivering the programme as part of your work day/routine?

a) Why do you feel this way?

2. What about

a) Time

b) Space

c) Privacy

d) Supervisors/in-charge support

e) Length of flip book session

**SECTION 2: PERCEIVED IMPACT OF BISCA**

1. Since completing your cessation sessions, have you managed to counsel any of your patients

to quit the use of tobacco?

If yes

What strategies did you use?

If no, why do you think that was?

**SECTION 3: STUDY PROCEDURES**

3.1. TRIAL RECRUITMENT

1. How did you learn about this study? What other ways could we tell people about the study?

Different places? Written or verbal information? Why is that a good idea?

2. What information about the study did you receive? How useful was it? How can we make it

more useful? What else do we need to say?

3.2. TRAINING

I would now like to ask you a few questions on the training on flipbook and counselling you were

offered before starting this project.

a. How do you feel about the training you received in delivering the behaviour

support?

b. What kinds of information and materials have been made available to you?

c. Did it help you to improve your knowledge and skills about delivering tobacco

cessation support?

2. How specifically did it help/which aspects of it helped?

a. Do you feel the need for any additional training on knowledge or skills?

3. If yes, what training would you like to receive?

4. Why would you like to receive this particular training?

(For those who didn’t deliver any session):

Why didn’t you deliver any sessions?

3. Is there anything else that you want to say?
