# Supplementary material for: Process evaluation of a pragmatic feasibility trial on smokeless tobacco cessation intervention delivered in dental hospitals
Source: BMC Public Health. 2024 May 16;24:1327. doi: 10.1186/s12889-024-18821-2 (PMC11100072; doi:10.1186/s12889-024-18821-2)
Supplement: Supplementary file 3 — Supplementary Material 3 [file 12889_2024_18821_MOESM3_ESM.docx]

Appendix 3. Fidelity index

| Adherence content | | | | | |
| --- | --- | --- | --- | --- | --- |
| Behavioural support intervention, Ingredients | Constituents BCTs | Implementation status | | | |
|  |  | Fully implemented | Partially implemented | Not implemented | Missing |
| Identify product | Assess current and past tobacco use behaviour |  | | | |
| Explain what the product contains | Provide information on consequences of  tobacco use |  | | | |
| Identify harmful ingredients | Provide information on consequences of  tobacco use  Salience of consequences |  | | | |
| Myths | Information about health consequences of  tobacco use.  Salience of consequences.  Re-attribution: elicit perceived causes of  behaviour and suggest alternative explanations |  | | | |
| Importance scale | Assess current readiness and ability to stop.  Identify reasons for wanting and not  wanting to stop tobacco use |  | | | |
| Benefits of stopping | Identify reasons for wanting and not  wanting to stop tobacco use  Framing/re-framing: Suggest the deliberate  adoption of a perspective on behaviour (e.g. its  purpose) in order to change cognitions or emotions  about performing the behaviour |  | | | |
| Assess past quit attempts | Assess past history of quit attempts.  Boost motivation and self-efficacy  Focus on past success |  | | | |
| Confidence scale | Assess current readiness and ability to stop  Focus on past success |  | | | |
| Help is available | Boost motivation and self-efficacy  Explain the importance of abrupt cessation  Explain expectations regarding treatment  programme  Focus on past success |  | | | |
| Social norms | Provide normative information about others  behaviour and experiences  Re-attribution |  | | | |
| Set quit date (preparation and  Planning) | Prompt commitment from the patient there  and then  Facilitate action planning/develop treatment  plan  Advise on environmental restructuring  Avoidance/reducing exposure to cues for the  behaviour  Instruction on how to perform behaviour |  | | | |
| Nicotine dependence and triggers  information | Inform about the nature of nicotine dependence (no  label)  Identify pattern of tobacco use (no label)  Facilitate goal setting  Facilitate barrier identification  Inform about antecedents |  | | | |
| Managing triggers | Facilitate action planning/develop treatment plan  Facilitate problem solving  Advise on changing routine  Behaviour substitution  Distraction: Advise to use alternative focus for attention to avoid triggers for chewing  Advise on setting graded task |  | | | |
|  | Self-incentive  Offer/direct towards appropriate written  materials |  | | | |
| Setting rewards and offering patient  booklet | Self-incentive  Offer/direct towards appropriate written materials |  | | | |
| Readiness scale | Assess current readiness and ability to stop  Framing and re-framing: Suggest that they  might think of quitting as reducing the risk of  consequences from continuing chewing (rather  than just the benefits of stopping) |  | | | |
| Strengthen ex-user identity | Strengthen ex-user identity  framing/re-framing: Encourage them to reframe in their mind why they wanted to stop in the first place  Encourage identification of self as a role model ‘who used to chew’:  Inform that their own behaviour may be an example to others |  | | | |
| Withdrawal symptoms information | Provide information on withdrawal  symptoms |  | | | |
| Managing withdrawls | Facilitate barrier identification and problem  solving  Facilitate action planning/develop treatment  plan  Facilitate goal setting  Behaviour substitution  Distraction  Self-talk (positive) |  | | | |
| Monitoring progress | Facilitate goal setting  Prompt self-recording |  | | | |
| Determine patients abstinence status | Boost motivation and self-efficacy |  | | | |
| Provide rewards | Self-rewards |  | | | |
| Discuss withdrawal symptoms | Assess withdrawal symptoms  Facilitate relapse prevention and coping |  | | | |

| Quality content | | |
| --- | --- | --- |
| Establish a positive , friendly and  professional relationship with the patient  and foster a sense that the patient’s experiences are understood | Build general Rapport |  |
| Give general reassurance to the patient  that his/her experiences are normal  and time limited | Provide reassurance |  |
| Prompt questions from the patient  answer clearly and accurately | Elicit and answer Questions |  |
| Adopt a style of interaction that involves listening carefully to the client and where appropriate reflecting back to the client key elements of what s/he is saying | Use reflective listening |  |
| Provide summary of information exchanged and establish a clear confirmation of decisions made and commitments entered into | Summarise info and confirm patients decisions |  |
| Use relevant information from the  Patient to tailor the behavioural support  provided/ flexible adaptation that takes  into account individual patient needs | Tailor interactions appropriately |  |
| Emphasise patient choice within the bounds of evidence based practice | Emphasise patient choice |  |
